# Acute COVID-19-Associated Cardiac Arrhythmia: A Case Series and Literature Review

**DOI:** 10.7759/cureus.27583

**Published:** 2022-08-01

**Authors:** Tsering Dolkar, Meet J Patel, FNU Jitidhar, Abubaker M Hamad, Amit Gulati, Muhammad H Dogar, Alix Dufresne

**Affiliations:** 1 Internal Medicine, OBH Interfaith Medical Center, Brooklyn, USA; 2 Medicine, Memorial Hospital of Converse County, Douglas, USA; 3 Internal Medicine, Maimonides Medical Center, Brooklyn, USA; 4 Internal Medicine/Cardiology, OBH Interfaith Medical Center, Brooklyn, USA; 5 Cardiology, OBH Interfaith Medical Center, Brooklyn, USA

**Keywords:** eliquis, cardiac arrythmia, atrial flutter rapid ventricular response, atrial fibrillation, covid 19

## Abstract

Coronavirus disease 2019 (COVID-19) is a highly contagious infectious disease caused by severe acute respiratory syndrome coronavirus 2 (SARS-CoV-2). We report two cases of COVID-19-associated atrial fibrillation (AF) in two elderly females and a case of atrial flutter (AFlutter) in a middle-aged male patient. We believe this case series will contribute to the literature on new-onset AF and AFlutter in patients with acute COVID-19 infection. This case series illustrates various case scenarios of patients developing cardiac arrhythmia with acute COVID-19 infection without any prior history or other explicable cause of AF/AFlutter. The exact mechanism behind COVID-19 infection leading to AF or AFlutter is still unknown. Of the three patients reported, two converted to sinus rhythm following medical management, and one did not convert to sinus rhythm despite medical treatment.

## Introduction

Coronavirus disease 2019 (COVID-19), caused by severe acute respiratory syndrome coronavirus 2 (SARS-CoV-2), is a highly contagious infectious disease. In the past two years, various studies have described the non-respiratory effects of COVID-19. We present a case series on COVID-19-associated cardiac arrhythmia. The patients in this case series developed new-onset cardiac arrhythmia concomitant with hospitalization due to COVID-19 infection.

## Case presentation

Case 1

The patient was a 64-year-old African-American male with a significant past medical history of hypertension, diabetes mellitus type 2, and unvaccinated to COVID-19 who presented to the ED with the complaint of shortness of breath for one week. He had started experiencing a dry cough and runny nose a week before the presentation. He had also started developing simultaneous lethargy and shortness of breath, which had worsened a day before the presentation. The patient had one episode of emesis containing only food particles without any blood before arriving at the hospital. He denied any recent travel history or exposure to any sick contacts. A review of his system was unremarkable, without any history of chest pain, palpitations, abdominal pain, diarrhea, sore throat, loss of smell, ear discharge, headache, or dizziness. The patient smoked cigarettes occasionally and denied using alcohol or any substance abuse.

**Figure 1:**
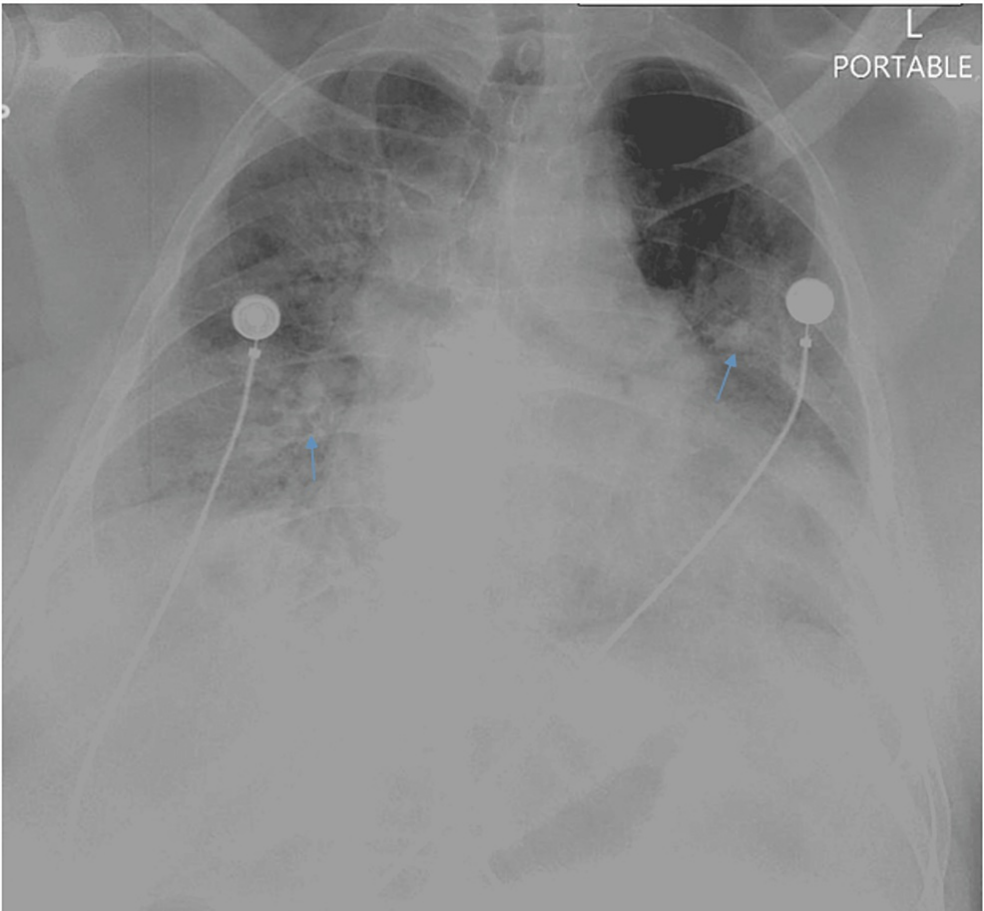
Chest X-ray on initial encounter showing bilateral airspace disease

In the ED, his vitals were as follows - blood pressure: 121/51 mmHg, pulse rate: 108 per minute, respiratory rate: 28 per minute, temperature: 98.6 °F, and oxygen saturation: 85% on room air. The patient was alert and oriented to time, place, and person on physical examination. He was sick-looking and tachypneic and had bilateral rales on chest auscultation. The rest of the physical examination was unremarkable. Chest X-ray (Figure 1) demonstrated bilateral diffuse airspace disease.

**Figure 2:**
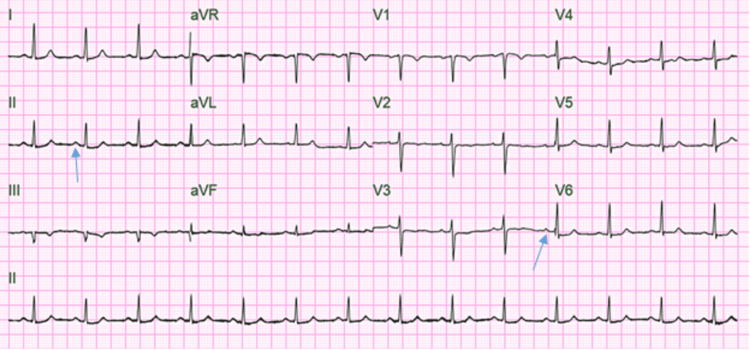
EKG on initial encounter showing normal sinus rhythm with HR of 84 bpm EKG: electrocardiogram; HR: heart rate

**Figure 3:**
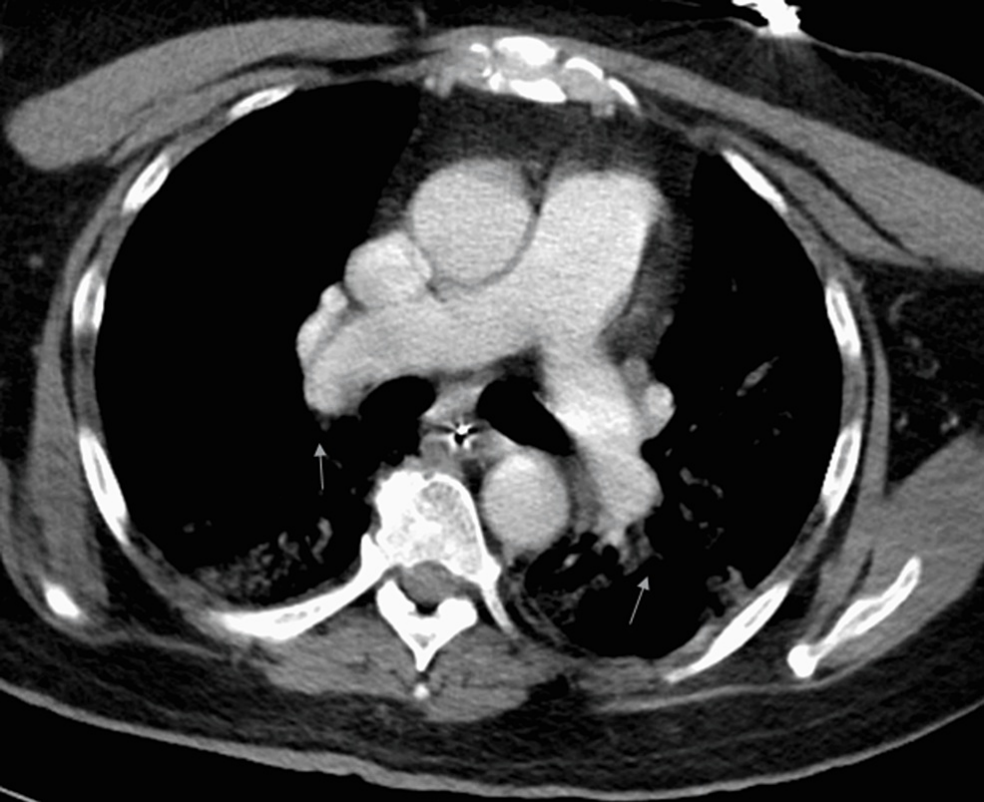
CT angiogram chest negative for pulmonary embolism CT: computed tomography

**Figure 4:**
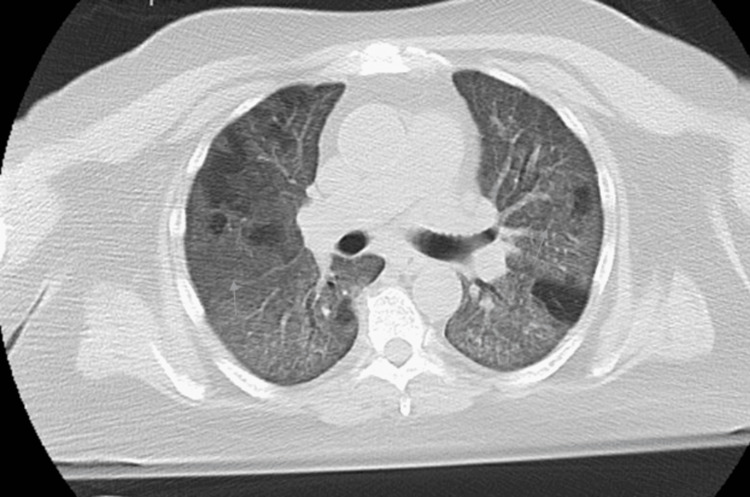
CT chest showing the ground-glass appearance of bilateral lungs CT: computed tomography

Electrocardiogram (EKG) was consistent with normal sinus rhythm with a heart rate (HR) of 84 beats per minute (bpm) (Figure 2). The SARS-CoV-2 PCR was positive. The patient was placed on a bilevel positive airway pressure (BiPAP) after the failure to maintain saturation on the nasal cannula. He was started on therapy with dexamethasone, azithromycin, and ceftriaxone. Initially, the treatment with remdesivir and tocilizumab was due to acute kidney injury and deranged liver function test, but after the initiation of dialysis, remdesivir and tocilizumab were infused. CT chest angiography (Figure 3) was negative for pulmonary embolism but demonstrated widespread ground-glass opacities (Figure 4). During the initial course of hospitalization, the patient was found to have worsening kidney function and low urine output. He developed seizures attributed to uremic encephalopathy versus hypoxic respiratory failure due to COVID-19. Urinary toxicology was negative. Troponin was elevated at 128 ng/L > 260 ng/L > 180 ng/L, attributed to end-stage renal disease (ESRD).* *The patient was intubated and placed on a mechanical ventilator. Hemodialysis was initiated, and he remained seizure-free during the rest of the hospital stay.

**Figure 5:**
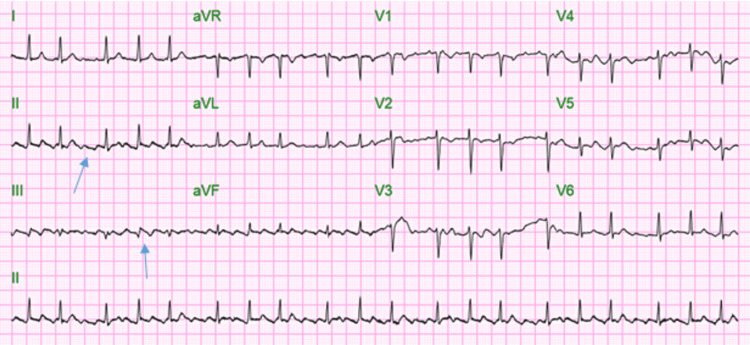
EKG on the 19th day of hospitalization showing atrial flutter with variable AV conduction with HR of 120 bpm EKG: electrocardiogram; HR: heart rate

On the 19th day of hospitalization, the patient developed acute AFlutter, as demonstrated on the telemetry monitor and confirmed with EKG (Figure 5). His HR fluctuated between 84-160 bpm at the time of AFlutter. The patient was started on diltiazem and amiodarone infusion. But the arrhythmia persisted throughout the hospitalization.

Baseline investigations (Table 1) on admission and on the day of arrhythmia did not show any significant differences. Thyroid-stimulating hormone (TSH) levels were normal.

**Table 1:** Laboratory investigations on the day of admission and on the day of arrhythmia WBC: white blood cells; BUN: blood urea nitrogen; BNP: brain natriuretic peptide; SARS-CoV-2: severe acute respiratory syndrome coronavirus 2; PCR: polymerase chain reaction; CRP: C-reactive protein; LDH: lactate dehydrogenase

Investigation	On admission	At the time of arrhythmia	Unit
Hemoglobin	9.4	8.1	g/dL
WBC	13.7	11.7	10^3^/uL
Platelets	195	110	10^3^/uL
Glucose	175	220	mg/dL
BUN	86.3	102.7	mg/dL
Creatinine	6.25	3.49	mg/dL
Sodium	136	133	mmol/L
Potassium	5.3	4.9	mmol/L
Chloride	105	101	mmol/L
Bicarbonate	19	24	mmol/L
Calcium	8.0	7.4	mg/dL
Albumin	3.0	2.1	g/dL
Corrected calcium	8.8	8.9	mg/dL
Magnesium	3.0	2.2	mg/dL
BNP	11.5	2003.7	pg/mL
SARS-CoV-2 PCR	Positive	Negative	
High-sensitivity troponin I	128	260	ng/L
D-dimer	6178	>7650	ng/mL
CRP	6.10	7.70	mg/dL
Ferritin	>5000	2110	ng/mL
LDH	906	1287	U/L

Brain natriuretic peptide (BNP) was elevated to 2003 pg/ml on the day of arrhythmia. Echocardiography (Echo) showed a normal left ventricular ejection fraction (LVEF) of 60% with Grade 2 diastolic dysfunction (DD) and no structural abnormality.

Anticoagulation was held due to acute GI bleed in the early course of hospitalization, but he was started on heparin infusion with close monitoring of activated partial thromboplastin time (aPTT) later during hospitalization. The patient succumbed to his illness and expired from acute respiratory distress syndrome (ARDS) due to COVID-19 infection after 32 days of hospitalization.

Case 2

A 71-year-old female patient, vaccinated for COVID-19, with a significant past medical history of hypertension and history of thyroidectomy was admitted due to dizziness of one-day duration. The patient denied other symptoms like chest pain, shortness of breath, palpitation, sweating, change in appetite, headache, abdominal pain, urinary complaints, fever, sore throat, or other significant issues.

The patient’s physical exam was unremarkable except for an irregular pulse, and she was awake, alert, and oriented to time, place, and person. Her glucose level on arrival was 133 mg/dL. Vital signs on arrival at the ED were as follows - blood pressure: 75/50 mmHg, pulse rate 93 per minute, respiratory rate: 16 per minute, temperature: 97.5 °F, and oxygen saturation: 95% on room air. Orthostatic vitals were negative. 

The patient’s SARS-CoV-2 PCR was positive. Baseline investigations on admission and later during the hospital course are presented in Table 2. TSH level was normal and urinary toxicology was negative. Troponin was negative, BNP was 212 pg/ml, and Echo was normal with LVEF of 65% with Grade 2 DD and no structural abnormality.

**Table 2:** Laboratory investigations on the day of admission and later in the course of hospitalization WBC: white blood cells; BUN: blood urea nitrogen; BNP: brain natriuretic peptide; SARS-CoV-2: severe acute respiratory syndrome coronavirus 2; PCR: polymerase chain reaction; CRP: C-reactive protein; LDH: lactate dehydrogenase

Investigation	On admission	Later course of hospitalization	Unit
Hemoglobin	10.9	8.6	g/dL
WBC	13.6	7.2	10^3^/uL
Platelets	355	365	10^3^/uL
Glucose	133	169	mg/dL
BUN	48.8	13.2	mg/dL
Creatinine	1.82	0.68	mg/dL
Sodium	137	139	mmol/L
Potassium	4.4	3.8	mmol/L
Chloride	103	104	mmol/L
Bicarbonate	19	26	mmol/L
Calcium	8.8	7.5	mg/dL
Albumin	3.2	2.1	g/dL
Corrected calcium	9.4	9.0	mg/dL
Magnesium	2.4	1.9	mg/dL
BNP	256	212	pg/mL
SARS-CoV-2 PCR	Positive	Negative	
High-sensitivity troponin I	14.9	8.8	ng/L
D-dimer	2017	1532	ng/mL
CRP	27	17.5	mg/dL
Ferritin	527	NA	ng/mL
LDH	134	NA	U/L

**Figure 6:**
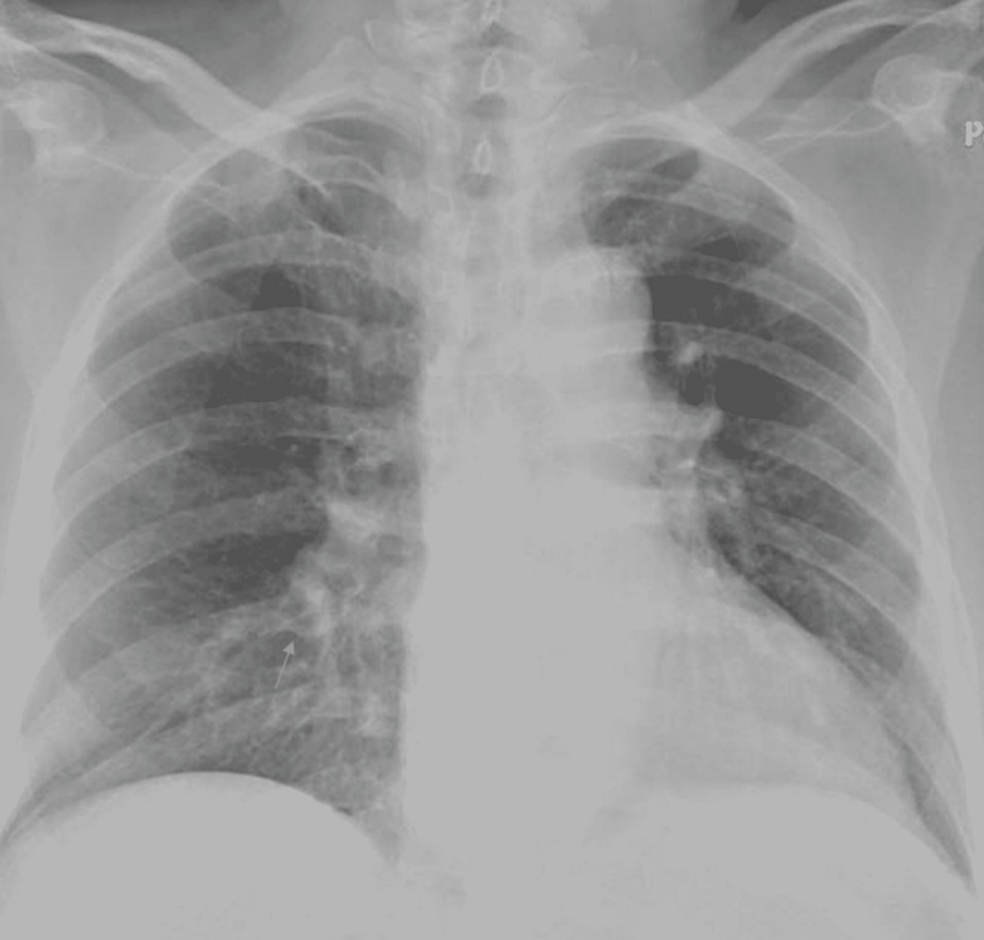
Chest X-ray showing a few infiltrates of the right lower lobe of the lung consistent with atelectasis

Chest X-ray showed right lower lobe infiltrates with atelectasis (Figure 6).

**Figure 7:**
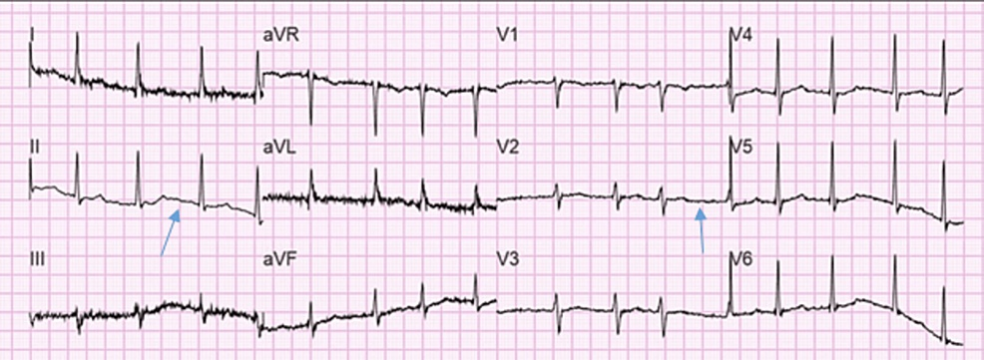
EKG showing atrial fibrillation with a controlled ventricular response with HR of 97 bpm EKG: electrocardiogram; HR: heart rate

**Figure 8:**
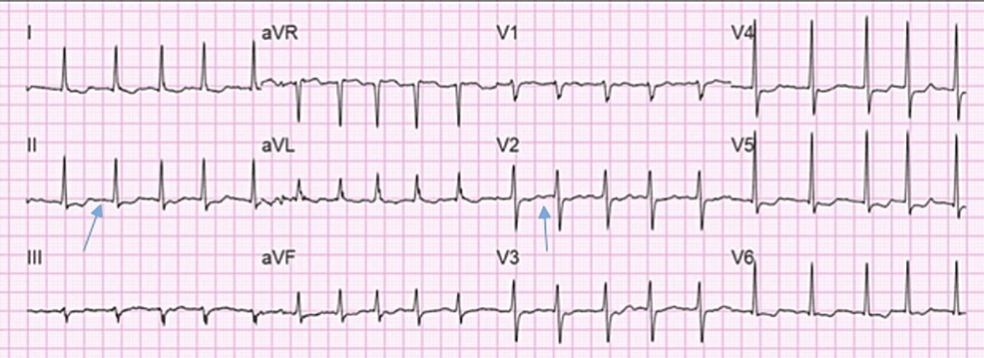
EKG showing atrial fibrillation with a rapid ventricular response with HR of 120 bpm EKG: electrocardiogram; HR: heart rate

EKG (Figure 7) showed AF with a controlled ventricular response, but a faster HR was observed later in the course of hospitalization (Figure 8). The patient’s HR was 93 bpm at the time of AF.

The patient's blood pressure improved with IV boluses. Subsequently, she was started on metoprolol, digoxin, and apixaban. After treatment, her HR was reduced to 85 bpm.

Case 3

A 66-year-old female patient with a past medical history of ESRD on hemodialysis three times a week via right arm AV fistula, hypertension, asthma, anemia of chronic disease, seizure disorder (not on any medication during admission), and unvaccinated to COVID-19 was admitted with complaints of fever and shortness of breath. The patient had spiked a fever during a dialysis session at her dialysis center and reported shortness of breath mostly on exertion for several days. She denied runny nose, cough, abdominal pain, headache, chest pain, joint pain, skin rashes, weight loss, alteration of bowel habits, or any urinary complaints.

On arrival at the ED, the vitals were as follows - blood pressure: 212/80 mmHg, pulse rate 96 per minute, respiratory rate: 18 per minute, temperature: 98.9 °F, and oxygen saturation: 100% on 5 liters via face mask. The physical examination was unremarkable. The patient was found to be SARS-CoV-2 PCR-positive. Infective markers such as WBC and lactate were normal, and blood cultures were negative. Baseline investigations on the day of admission and on the second day of hospitalization are presented in Table 3. TSH was normal, and urinary toxicology was negative. Troponin was elevated at 131 ng/L, attributed to ESRD. BNP was elevated at 2964 pg/ml. Echo showed a normal LVEF of 55% with Grade 2DD and mild mitral regurgitation.

**Table 3:** Laboratory investigations on the day of admission and on the second day WBC: white blood cells; BUN: blood urea nitrogen; BNP: brain natriuretic peptide; SARS-CoV-2: severe acute respiratory syndrome coronavirus 2; PCR: polymerase chain reaction; CRP: C-reactive protein; LDH: lactate dehydrogenase

Investigation	On admission	Second day of hospitalization	Unit
Hemoglobin	7.8	9.0	g/dL
WBC	4.3	4.5	10^3^/uL
Platelets	133	196	10^3^/uL
Glucose	96	97	mg/dL
BUN	12.7	10.2	mg/dL
Creatinine	4.35	3.14	mg/dL
Sodium	142	141	mmol/L
Potassium	4.0	3.7	mmol/L
Chloride	99	97	mmol/L
Bicarbonate	29	31	mmol/L
Calcium	8.1	8.7	mg/dL
Albumin	3.5	4.0	g/dL
Corrected calcium	8.5	NA	mg/dL
Magnesium	2.0	2.0	mg/dL
BNP	2964	NA	pg/mL
SARS-CoV-2 PCR	Positive	NA	
High-sensitivity troponin I	131.3	98.3	ng/L
D-dimer	651	518	ng/mL
CRP	12.9	4.0	mg/dL

**Figure 9:**
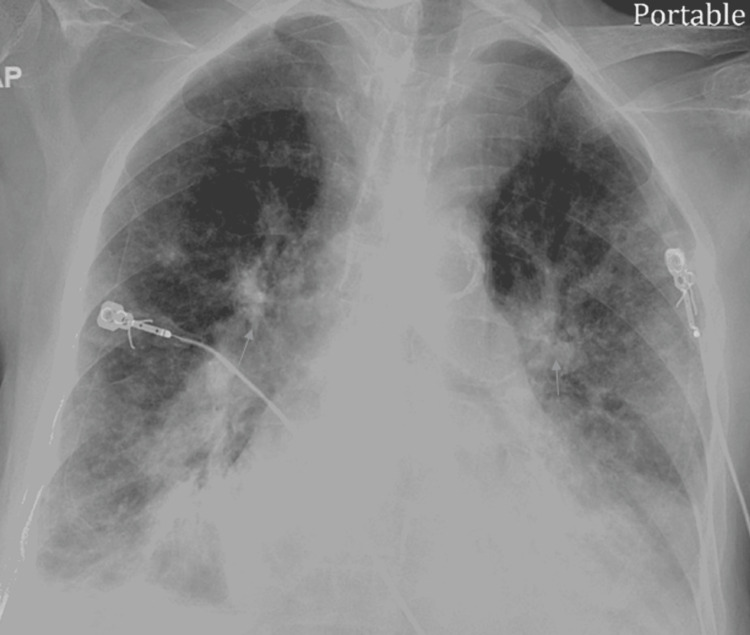
Chest X-ray showing bilateral perihilar opacities with small pleural effusions

The patient had a normal echocardiogram. Chest X-ray (Figure 9) showed bilateral perihilar opacities with small pleural effusions, which had been present a year ago as well. Hypertensive urgency was treated with hydralazine IVP, which later resolved, and the patient was resumed on nifedipine.

**Figure 10:**
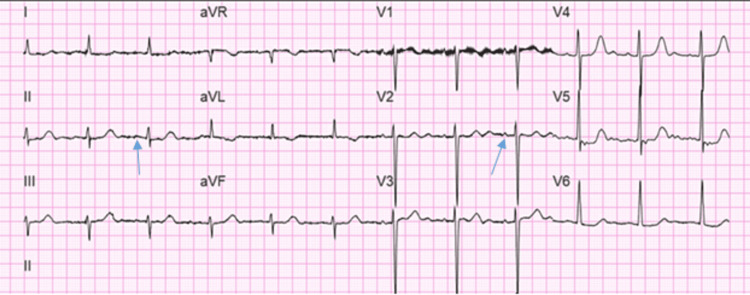
EKG showing normal sinus rhythm on initial encounter with HR of 69 bpm EKG: electrocardiogram; HR: heart rate

**Figure 11:**
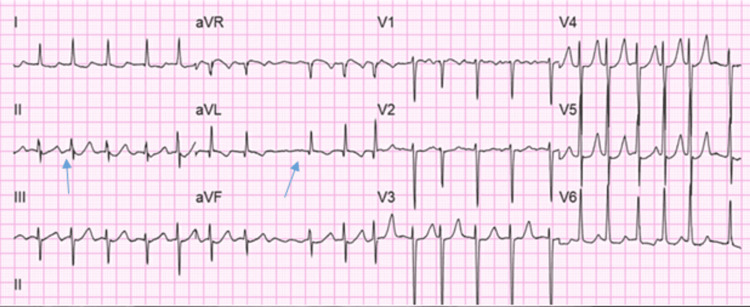
EKG showing atrial fibrillation with HR of 121 bpm on the second day of hospitalization EKG: electrocardiogram; HR: heart rate

The patient’s EKG (Figure 10) showed a normal sinus rhythm during the initial encounter. However, she developed AF with HR of 121 bpm on her second day of hospitalization, as evident on EKG (Figure 11). The patient received Cardizem 10 mg IV push and metoprolol 25 mg PO twice daily. She converted to sinus rhythm with HR of 84 bpm on the same day without any further intervention. The patient was treated for COVID-19 infection with dexamethasone.

## Discussion

Emerging evidence shows that COVID-19 infection can lead to hypercoagulable states associated with macrovascular complications and is also responsible for the pathogenesis of ARDS [1]. Many reports show an association of COVID-19 with autoimmune and autoinflammatory conditions [2,3]. A few studies have also shown the presence of new-onset cardiac arrhythmias in patients with COVID-19 infection.

The incidence of AF is high in patients with severe pneumonia, ARDS, and sepsis. Some studies show that 23-33% of seriously ill patients with sepsis or ARDS have AF recurrences, and 10% develop new-onset AF [4]. Based on the existing literature, the incidence of AF with COVID-19 infection is reportedly 19-21% [5]. Further studies are needed to confirm the high risk of developing AF to provide treatment optimization in patients with COVID-19 [6]. We present a case series on patients developing new-onset AF/AFlutter after developing severe COVID-19 infection. This case series will add to the existing literature on new-onset AF/AFlutter in patients with COVID-19. Earlier reported cases on the association of COVID-19 with new-onset AF involved two middle-aged male patients [7]. We report two cases of COVID-19-associated AF in two elderly females and one case of AFlutter in a middle-aged male patient.

The three cases we presented depicted various scenarios of patients developing cardiac arrhythmia with acute COVID-19 infection without any prior history or other causes of AF. The mechanisms behind COVID-19 infection leading to AF are poorly understood. The proposed mechanisms include increased inflammation, electrolyte abnormalities, acid-base abnormalities, and endothelial damage, which all lead to myocardial tissue damage and increased sympathetic activity, leading to increased susceptibility to AF. The pathology causing arrhythmia may include reduced angiotensin-converting enzyme 2 (ACE2) receptor availability, CD147- and sialic acid-spike protein interaction, and enhanced inflammatory signaling [8].

Telemonitoring and personal EKG devices could facilitate the detection of arrhythmia in COVID-19 patients for the optimization of care [9]. In a multicentric global survey involving 1100 EP professionals regarding hospitalized COVID-19 patients, various arrhythmic manifestations were observed, ranging from benign to potentially life-threatening ones [10]. Several electrocardiographic features have been reported in COVID-19 patients, not limited to arrhythmias [11].

We presented three diverse case scenarios of acute COVID-19-associated cardiac arrhythmia. In all three cases, TSH was normal and urine drug screen was negative. There were no signs of alcohol withdrawal. Infective markers such as WBC and lactate were normal. Elevated troponin levels in cases 1 and 3 were attributed to ESRD. Echo in all three cases showed no structural abnormalities and no atrial enlargements. None of the cases was paroxysmal in nature as all three patients had sustained arrhythmia requiring treatment. All three cases showed sinus rhythm on admission with no history of cardiac arrhythmias. They developed new-onset AF/AFlutter later in the course of hospitalization. Case 1 was critically ill with severe COVID-19 infection and eventually succumbed to the disease and expired. Cases 2 and 3 converted to sinus rhythm before discharge. The precise mechanism behind AF is still unknown. Elderly patients with existing heart complaints are at a higher risk of developing new-onset AF associated with acute COVID-19 infection [12], as seen in our cases.

## Conclusions

The literature on the association between COVID-19 infection and cardiac arrhythmia continues to evolve. New-onset cardiac arrhythmia in patients with COVID-19 infection has acquired great interest in the medical community. Long-term repercussions of COVID-19 illness-induced cardiac arrhythmia are still unknown. A larger, more extensive study needs to be conducted on patients with new-onset cardiac arrhythmia in the context of COVID-19 infection to assess the disease's real impact and define the arrhythmic manifestations in COVID-19 infections.
